# Exploring source differences on diet-tissue discrimination factors in the analysis of stable isotope mixing models

**DOI:** 10.1038/s41598-020-73019-x

**Published:** 2020-09-25

**Authors:** Wilbert T. Kadye, Suzanne Redelinghuys, Andrew C. Parnell, Anthony J. Booth

**Affiliations:** 1 Department of Ichthyology and Fisheries Science, Rhodes University, P.O. Box 94, Grahamstown/Makhanda, 6140 South Africa; 2 Hamilton Institute, Insight Centre for Data Analytics, Maynooth University, Kildare, Ireland

**Keywords:** Ecology, Zoology

## Abstract

Stable isotope mixing models are regularly used to provide probabilistic estimates of source contributions to dietary mixtures. Whilst Bayesian implementations of isotope mixing models have become prominent, the use of appropriate diet-tissue discrimination factors (DTDFs) remains as the least resolved aspect. The DTDFs are critical in providing accurate inferences from these models. Using both simulated and laboratory-based experimental data, this study provides conceptual and practical applications of isotope mixing models by exploring the role of DTDFs. The experimental study used Mozambique Tilapia *Oreochromis mossambicus*, a freshwater fish, to explore multi-tissue variations in isotopic incorporation patterns, and to evaluate isotope mixing model outputs based on the experiment- and literature-based DTDFs. Isotope incorporation patterns were variable for both muscle and fin tissues among the consumer groups that fed diet sources with different stable isotope values. Application of literature-based DTDFs in isotope mixing models consistently underestimated the dietary proportions of all single-source consumer groups. In contrast, application of diet-specific DTDFs provided better dietary estimates for single-source consumer groups. Variations in the proportional contributions of the individual sources were, nevertheless, observed for the mixed-source consumer group, which suggests that isotope assimilation of the individual food sources may have been influenced by other underlying physiological processes. This study provides evidence that stable isotope values from different diet sources exhibit large variations as they become incorporated into consumer tissues. This suggests that the application of isotope mixing models requires consideration of several aspects such as diet type and the associated biological processes that may influence DTDFs.

## Introduction

Stable isotope mixing models are important tools in trophic ecology studies to quantitatively estimate the composition of consumer diets^1–4^. Recently, Bayesian inference-based isotope mixing models have risen to prominence in providing robust inferences on consumer diets by addressing challenges such as the occurrence of multiple prey sources in food webs^5,6^, the uncertainties associated with measurement, source and mixture process errors^1,7,8^, the incorporation of concentration dependences^9^ and the use of prior information for both sources and mixtures^10–12^. Despite these significant improvements, the use of appropriate diet-tissue discrimination factors, which have a direct influence on the correct interpretation of mixing models outputs, remains a key challenge^8,13^. Addressing this challenge is critical because diet-tissue discrimination factors are a major source of uncertainty in mixing models, and isotope mixing models are extremely sensitive to these factors^13,14^.

Diet-tissue discrimination factors (DTDFs), represented as $\Delta {}^{13}C$ for the carbon and $\Delta {}^{15}N$ for the nitrogen stable isotopes (the most frequently used isotopes in trophic ecology) reflect the amount of change in dietary stable isotope values when they become incorporated into consumer tissues^15^. This diet-to-tissue difference may be a consequence of preferential elimination and use of lighter isotopes in metabolic pathways, which result in progressive accumulation and enrichment of the unused heavier isotopes in consumer tissues^16^. For example, animal tissues may become progressively enriched in the heavier carbon isotope $^{13} C$ relative to their diets due to either isotopic kinetic effects in anabolic pathways that result in the accumulation of this heavy isotope^17^ or due to the preferential use and elimination of lighter carbon isotope $^{12} C$ in catabolic pathways^18,19^. Similarly, the heavier nitrogen isotope ${}^{15}N$ may become progressively enriched in animal tissues relative to their diets due to preferential removal of the lighter nitrogen isotope ${}^{14}N$, such as during deamination and transamination of amino acids with freely exchangeable nitrogen^20^ in order to produce isotopically light metabolites^15,21,22^. Alternatively, diet-to-tissue isotopic differences may be a consequence of isotope routing, which may result in unequal allocation of isotopes among different body tissues during synthesis of macromolecules^23,24^.

Obtaining accurate DTDFs that can be used in mixing models usually requires empirical experiments in which animals are kept in captivity and fed isotopically distinct diets over a lengthy period. Several such empirical studies have been conducted to validate DTDFs and the associated inter- and intra-specific isotope turnover rates^16,25–27^. These studies have shown that following a switch to diets with known stable isotope values, different species and different body tissues exhibit variation in both isotope-to-tissue turnover rates and the diet-to-tissue isotopic differences because of the different catabolic and anabolic pathways that are involved during growth and metabolism. These patterns have been illustrated in several animal taxa, including mammals^28,29^, birds^30,31^, reptiles^32^, amphibians^33^ and invertebrates^34^. Similarly, in fishes, studies have demonstrated interspecific differences in diet-to-tissue isotope assimilation due to differences in diet quality, feeding and excretion rates^35,36^, and among different body tissues, with metabolically active tissues, such as blood, liver and fins, showing relatively faster turnover rates compared to less active and structural tissues, such as muscle, scales and otoliths^19,37,38^.

Although empirical studies on DTDFs and isotope turnover rates have been crucial in understanding the dynamics of isotope incorporation in animal tissues^24,39^, the use of this information in isotope mixing models remains limited^40–42^, in part, because such information is either unavailable for a wide range of taxa or its acquisition may be costly and impractical. Consequently, many studies on mixing models rely on proxy DTDFs that are derived either from previous studies (e.g.^17,21^) or from closely related taxa (e.g.^25,43,44^). The use of proxy DTDFs in isotope mixing models has, nonetheless, often raised concern due to the potential errors in estimating prey source contribution, prompting calls to use more accurate DTDFs^8,29^.

In freshwater ecosystems, the increasing interest in the application of isotope mixing models^45^, where many such studies rely on literature-based DTDFs (e.g.^46,47^), highlights the need to explore the use of experimentally-derived DTDFs that are informative. Recent studies on freshwater fishes based on both laboratory^36,42^ and field^40,41^ research have already shown that variations in DTDFs for both $\delta^{13} C$ and $\delta {}^{15}N$ isotopes have implications for interpreting food webs. The present study, therefore, explores the utility of isotope mixing models, and how their interpretation can be influenced by the use of either literature-based or empirically-derived diet-tissue discrimination factors. Using Mozambique Tilapia *Oreochromis mossambicus* (Peters 1852), a freshwater cichlid fish that is native to east coastal rivers of southern Africa^48^, as a case study, our research addressed two objectives. First, following the recent observations in empirical studies on stable isotope feeding trials on different fish taxa (e.g.^36,38,42,49^), our study explored the variation in both diet sources with different stable isotope values and different body tissues in relation to stable isotope incorporation and diet-to-tissue discrimination patterns. Specifically, the present study hypothesized that isotope incorporation rates and DTDFs would vary both for diet sources with different $\delta^{13} C$ and $\delta {}^{15}N$ values and for different body tissues of the study species. These patterns were evaluated using the classic isotope incorporation and diet-to-tissue discrimination models based on the Bayesian approach to estimate joint posterior distributions of model parameters. Second, our study predicted that using experimentally-derived DTDFs, together with including the uncertainties of such factors in Bayesian-based isotope mixing models (e.g.^8,12^) would enhance the inference on consumer diet source estimates.

## Materials and methods

### Ethics statement

Permission for the research was granted by Eastern Cape’s Department of Economic Development and Environmental Affairs through permit number CRO 190/16CR. Experimental procedures and ethical guidelines were reviewed and approved by the Rhodes University Ethics Committee (RUEC), with the care of animals being guided by the South African National Standards (SANS) 10386:2008.

### Isotope mixing models conceptual framework

**Figure 1:**
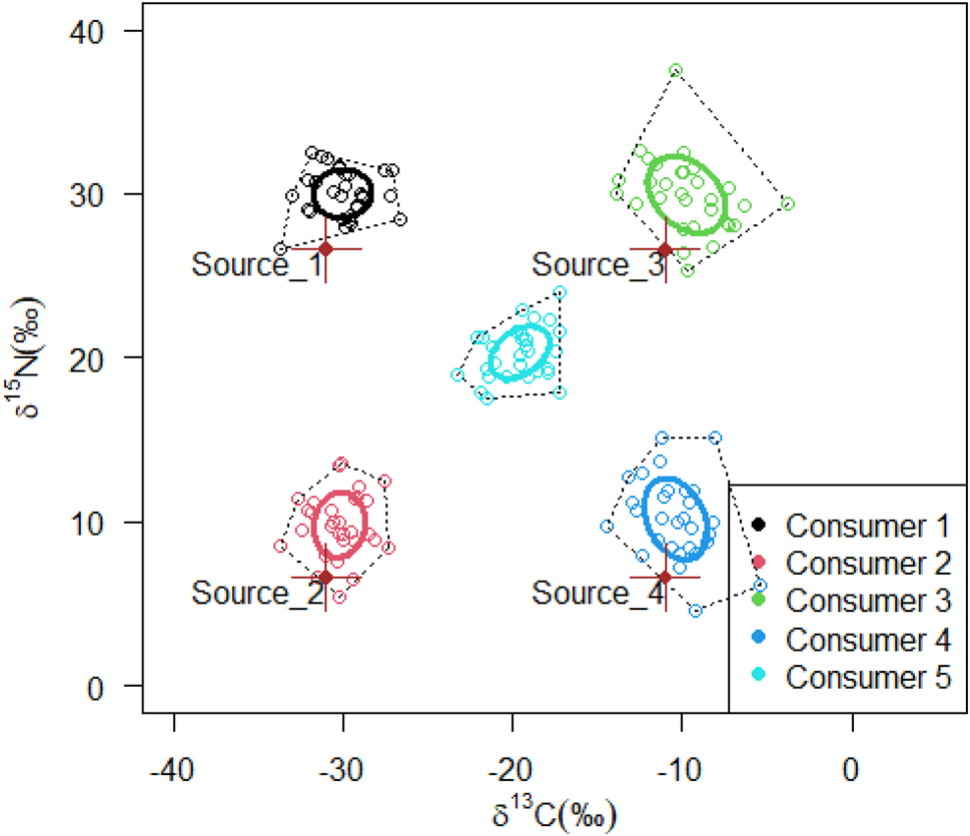
Simulated four diet sources and five consumer groups together with their sample size-corrected standard ellipse area (SEA_c_). The values for the diet sources include the mean and standard deviations for $\delta^{13} C$ and $\delta {}^{15}N$. The consumer groups comprise convex hulls, which encompass all individuals, and SEA_c,_ which encompass 40% of the sample.

Firstly, the present study illustrated the application of isotope mixing models in estimating dietary composition of different consumer groups using simulated data that were consistent with the experimental case study below (see the R script in Supplementary 1). The simulated data comprised five consumer groups and four diet sources that were each distinguished by having different $\delta^{13} C$ and $\delta {}^{15}N$ values. Four of the hypothetical consumer groups were single-source groups, with each consumer group assumed to feed on one specific hypothetical diet source. In contrast, the fifth consumer group was a mixed-source group that was assumed to feed on all four hypothetical diet sources (Fig. 1). The five consumer groups were assumed to have similar diet-tissue discrimination for all diets. Diet-tissue $\Delta {}^{13}C$ and $\Delta {}^{15}N$ values of 1.0 ± 2.0‰ and 3.4 ± 2.0‰, respectively, were used in this study. These were consistent with literature-based values^25,50^ that are widely used in isotope mixing models studies. To test whether isotope mixing models accurately reflected the dietary composition of the designated hypothetical consumer groups, either as dietary specialists (consumers 1 to 4) or as dietary generalists (consumer 5), a Bayesian-framework based mixing model *MixSIAR*^51^ was applied using the R statistical program.

Secondly, using Mozambique Tilapia *O. mossambicus* as a case study, we conducted a separate empirical experiment to (1) evaluate variation in both isotopic incorporation patterns and diet-tissue discrimination factors based on multiple diets for muscle and fin tissues, and (2) apply the isotope mixing model on the experimental data to evaluate the most appropriate DTDFs in estimating dietary composition. This experiment was designed to provide data that deviated from the hypothetical framework. This facilitated the evaluation of isotopic incorporation patterns for both multiple diets and different body tissues together with evaluating appropriate DTDFs for the isotope mixing models. Multi-tissue comparisons have wider applications in the trophic ecology of fishes. For example, fin tissues are often used to either reveal different temporal patterns associated with isotope assimilation^37,52^ or as non-lethal alternatives to muscle tissue in food web studies^53^. Consistent with the simulated data, the experiment comprised five consumer groups of fish that were fed diet sources with different stable isotope compositions. Four of these experimental groups (consumers 1 to 4) were designated as single-source consumer groups because they were all fed single diet sources that corresponded with their respective groups. The fifth consumer group (consumer 5) was designated as the mixed-source group because it was fed an equal mix of the four sources. The mixing models were then applied to compare whether using DTDFs would provide better estimates of the dietary composition for fish that were single sources (dietary specialist) and those that were fed multiple sources (generalist consumer group). To achieve this, this study compared isotope mixing model outputs that were obtained using diet-tissue discrimination factors from literature and diet-specific discrimination factors that were derived from the feeding trial experimental study.

### Experimental design and sample collection

In March 2016, 150 wild-caught individuals of Mozambique Tilapia *O. mossambicus* were transported to the Freshwater Ecology Laboratory at the Department of Ichthyology and Fisheries Science, Rhodes University, South Africa. The fish were captured by seine netting, and transported in an aerated 200 L tank. They ranged in size from 50 to 125 mm in standard length. At the laboratory, fish were transferred into four aquaria, each measuring 90 cm × 32 cm × 35 cm in length, width and height, respectively. Water quality in the aquaria was maintained by an air circulation filtering mechanism. Water temperature was maintained at 20 °C and dissolved oxygen was kept at saturation level. The laboratory had a time-controlled illumination cycle of a 12 h day-night photoperiod. The fish were weaned onto commercial fish flakes ($\delta^{13} C$ = − 23.5 ± 0.2‰, $\delta {}^{15}N$ = 7.8 ± 0.2‰), and were fed ad libitum for 60 days to acclimate to laboratory conditions. The 60-day acclimation period was based on a pilot experiment that indicated comparable stable isotope values among experimental animals.

Five pelletized experimental diet sources (sources 1 to 5) were created in the laboratory for the experiment. Four of these sources were formulated using ingredients with different composition of carbohydrates, proteins, and lipids, and were all isocaloric and isonitrogenous, whereas the fifth source was an equal mix of the four sources. Each diet source comprised a total of 1 kg of different ingredients, using either fishmeal or soya as protein sources, and either maize or rice as carbohydrate sources, which resulted in different $\delta^{13} C$ and $\delta {}^{15}N$ values for the different diets (Table 1). Sunflower oil was added to sources 2, 3 and 4, as a lipid source and binder, whereas source 1 contained fish oil from the fish meal. All diet sources contained vitamix to supplement essential vitamins and minerals. Source 1 was distinguished by high carbon ($\delta^{13} C$ = − 14.7 ± 0.01‰), whereas source 2 had the highest nitrogen ($\delta {}^{15}N$ = 10.8 ± 0.06‰) stable isotope values (Table 1). Sources 3 and 4 had low nitrogen isotope values ($\delta {}^{15}N$ = 1.4 $\pm$ 0.52‰ and $\delta {}^{15}N$ = 0.8 $\pm$ 0.03‰, respectively), whereas source 5, which was a mix of the four sources, had intermediate carbon ($\delta^{13} C$ = − 21.9 $\pm$ 0.76‰) and nitrogen ($\delta {}^{15}N$ = 4.5 $\pm$ 0.83‰) isotope values.

**Table 1:** Ingredient composition and weight (grams) for each of the formulated experiment diet sources, their protein and energy content, and their δ^13^C and δ^15^N isotope (‰) values.

	Source 1	Source 2	Source 3	Source 4	Source 5
Fishmeal (g)	461	455.8	0	0	229.2
Soya (g)	0	0	632.7	643.7	315.2
Maize (g)	536.8	0	333.4	0	217.5
Rice (g)	0	526	0	289.2	213.5
Sunflower oil (g)	0	16	31.7	64.9	22.3
Vitamix (g)	2.2	2.2	2.2	2.2	2.2
Protein (%)	35.0	35.0	35.0	35.0	35.0
Protein digestible (%)	30.2	30.2	33.6	33.7	32.0
Energy level (MJ/kg)	18.7	18.7	18.7	18.7	18.7
Energy level (kcal/kg)	4477.7	4476.6	4476.6	4476.6	4476.9
$\delta {}^{13}C$ (‰)	− 14.7 ± 0.01	− 23.5 ± 0.02	− 22.9 ± 0.06	− 26.8 ± 0.08	− 21.9 ± 0.76
$\delta {}^{15}N$ (‰)	9.9 ± 0.26	10.8 ± 0.06	1.4 ± 0.52	0.8 ± 0.03	4.5 ± 0.83

After the 60 days acclimation period, fish were transferred to experimental tanks, each measuring 30 cm × 23 cm × 24 cm in length, width and height, respectively. Each experimental tank contained an undergravel bed with an air-lift oxygenation system. The five experimental diet treatments were randomly assigned to 25 experimental tanks that were arranged in sequence. Therefore, each treatment diet source had five replicate tanks, with each tank holding a maximum of six individual fish, and all fish were fed ad libitum daily. The tanks were siphoned daily to remove faecal matter and uneaten food, and a minimum of 50% water was exchanged.

On day 0, three fish were sampled to establish initial values for $\delta^{13} C$ and $\delta {}^{15}N$ for muscle and fin tissues. To monitor the change $\delta^{13} C$ and $\delta {}^{15}N$ for both muscle and fin tissues, fish were subsequently sampled 10, 20, 30, 40 50, 60, 90 and 120 days after the diet switch. On each sampling occasion, three fish were randomly selected from each of the five treatment diets. Fish were euthanised using a lethal dose of 2-phenoxyethanol, after which each fish was measured for total and standard length (mm), and weight (g). From each of the 15 fish that were sampled during each occasion, muscle and caudal fin tissue samples were collected for stable isotope analysis. Muscle tissue samples were cut below the dorsal fin, and fin samples were taken from the upper lobe of the caudal fin tissue. Samples of muscle and fin tissue were oven-dried at 60 °C for a maximum of 72 h, after which they were ground to a fine powder using a mortar and pestle.

Isotopic analysis was conducted on a Flash EA 1112 Series coupled to a Delta V Plus stable light isotope ratio mass spectrometer via a ConFlo IV system (all equipment supplied by Thermo Fischer, Bremen, Germany), housed at the Stable Isotope Laboratory, Mammal Research Institute, University of Pretoria, South Africa. Aliquots of approximately 0.6 to 0.65 mg were weighed into tin capsules that were pre-cleaned in toluene. Two laboratory running standards (Merck Gel: $\delta^{13} C$ = − 20.26 $\pm$ 0.07‰, $\delta {}^{15}N$ = 7.89 $\pm$ 0.07‰, C% = 41.28, N% = 15.29, and DL-Valine: $\delta^{13} C$ = − 10.57 $\pm$ 0.06‰, $\delta {}^{15}N$ = − 6.15 $\pm$ 0.06‰, C% = 55.50, N% = 11.86) were used to evaluate the precision of the isotopic composition. Stable isotope ratios, $\delta^{13} C$ and $\delta {}^{15}N$, were determined in parts per thousand (‰) relative to Vienna Pee Dee Belemnite and atmospheric air standards, respectively, and according to the formula: $\delta^{13} C$ and $\delta {}^{15}N$ = R_sample_/R_standard_ − 1, where R = ^13^*C*/^12^*C* or ^15^*N*/^14^*N*.

### Isotope incorporation models and diet-tissue discrimination factors

To examine isotope incorporation patterns of the single-source groups for both muscle and fin tissues, the classic one- and two-compartment models^24,31^ were fitted. One-compartment models assume that substrate metabolism during isotope incorporation process is governed by first-order kinetics, whereas two- or multiple-compartment models assume that isotope incorporation is governed by two or multiple phases, each depicting a distinct compartment that is loosely referred to as a “pool”^22,54^. The isotopic incorporation models were assumed as:

$$\delta X_{i} = f(t_{i} ,\theta ) + \varepsilon_{i} ,$$

where $\delta X_{i}$ is the isotopic value of a given element (either $\delta^{13} C$ or $\delta^{15} N$) for a given tissue at time $t$, $\theta$ is a vector of unknown model parameters, $\varepsilon_{i} \sim N(0,\sigma^{2} )$ is the independent random error of $\delta X_{i}$, and $f(t_{i} ,\theta )$ is either the one- or the two-compartment model. Similar to other studies, the one- and two compartment models were thus fitted using the following equations:

$$f_{1} (t_{i} ,\theta_{1} ) = \delta X_{\infty } - (\delta X_{\infty } - \delta X_{0} )e^{{ - \tfrac{t}{(1/\lambda )}}} ,$$

where $$\theta_{1} = (\delta X_{0} ,\delta X_{\infty } ,\lambda ),$$

and

$$f_{2} (t_{i} ,\theta_{2} ) = \delta X_{\infty } - (\delta X_{\infty } - \delta X_{0} ) \times (pe^{{ - \tfrac{t}{{(1/\lambda_{1} )}}}} + (1 - p)e^{{ - \tfrac{t}{{(1/\lambda_{2} )}}}} ),$$

where $$\theta_{2} = (\delta X_{0} ,\delta X_{\infty } ,\lambda_{1} ,\lambda_{2} ,p)$$.

In both equations, the isotopic value of a given element (either $\delta^{13} C$ or $\delta {}^{15}N$) for a given tissue at time *t* is based on pre- $(\delta X_{0} )$ and post-switch $(\delta X_{\infty } )$ isotope values. The average residence time $(\tau )$ was estimated as the reciprocal of the fractionation incorporation rate ($\tau = 1/\lambda$)^19,37^. For two-compartment models, $p$ refers to the fractional size of each pool, such that $\sum\nolimits_{i} {p_{i} }$ = 1^55^, and the average residence time is estimated as $\tau_{mean} = p\tau_{1} + (1 - p)\tau_{2}$^19^. Thus, the two models are nested, with model 1 being equivalent to setting $p$ = 1 in model 2.

Bayesian estimators for the isotope incorporation models were used to construct the joint posterior probability distributions of the model parameters. The posterior distributions together with the likelihood and prior probabilities for the one- and two-compartment models were given as:

$$\begin{gathered} p(\delta X_{\infty } ,\delta X_{0} ,\lambda ,\sigma_{\delta X}^{2} |\delta X) \propto \prod\limits_{i = 1}^{n} {\mathcal{L}(\delta X_{i} |\delta X_{\infty } ,\delta X_{0} ,\lambda ,\sigma_{\delta X}^{2} )} \hfill \\ \quad \quad \quad \times \pi (\sigma_{\delta X}^{2} )\pi (\delta X_{\infty } )\pi (\delta X_{0} )\pi (\lambda ), \hfill \\ \end{gathered}$$

and

$$\begin{gathered} p(\delta X_{\infty } ,\delta X_{0} ,\lambda_{1} ,\lambda_{2} ,p,\sigma_{\delta X}^{2} |\delta X) \propto \prod\limits_{i = 1}^{n} {\mathcal{L}(\delta X_{i} |\delta X_{\infty } ,\delta X_{0} ,\lambda_{1} ,\lambda_{2} ,p,\sigma_{\delta X}^{2} )} \hfill \\ \quad \quad \quad \times \pi (\sigma_{\delta X}^{2} )\pi (\delta X_{\infty } )\pi (\delta X_{0} )\pi (\lambda_{1} )\pi (\lambda_{2} )\pi (p). \hfill \\ \end{gathered}$$

In both equations, $p$ is the posterior probability, $\sigma^{2}$ is the variance, $\mathcal{L}$ is the likelihood function and $\pi$ is the prior distribution. The models were run with three Markov chains and 100,000 iterations per chain, 50,000 burn-ins and a thinning interval of 50. The analysis was done using the R package *rjags*^56^. Prior distributions for model parameters were specified based on normal distributions with a mean and variance ($dnorm(\mu ,\sigma^{2} )$) for $\delta X_{0}$, $\delta X_{\infty }$ and $\lambda$. Informative priors were used and these were specified based on the observed incorporation patterns from the data (Table 2). The deviance information criterion (*DIC*) was used as the Bayesian alternative of Akaike’s information criterion (*AIC*) to compare model fit between one- and two-compartment models. Comparative non-linear least squares (NLS) models were fitted based on the Levenberg–Marquardt algorithm using the R package *minpack.lm*^57^ (see Supplementary 2). For the NLS models, sample size-corrected Akaike’s information criterion ($AICc$)^31^ were used to evaluate the relative importance of one- and two-compartment model (Supplementary 2, Table S1).

**Table 2:** Informative priors (indicating the mean and standard deviation) for the Bayesian isotope incorporation model parameters and for the diet-to-tissue discrimination factors (DTDFs) that were used in isotope mixing models.

Diet source	Parameter	$\delta {}^{13}C$		$\delta {}^{15}N$	
		Muscle	Fin	Muscle	Fin
1	$\delta X_{\infty }$	− 19, 10	− 17, 10	10, 10	12, 10
	$\delta X_{0}$	− 23, 10	− 21, 10	9, 10	9, 10
	$\lambda$	0.1, 5	0.1, 5	0.1, 5	0.1, 5
2	$\delta X_{\infty }$	− 20, 10	− 18, 10	11, 10	12.5, 10
	$\delta X_{0}$	− 23, 10	− 21, 10	9, 10	9, 10
	$\lambda$	0.1, 5	0.1, 5	0.1, 5	0.1, 5
3	$\delta X_{\infty }$	− 20, 10	− 18, 10	9, 10	7, 10
	$\delta X_{0}$	− 23, 10	− 21, 10	9, 10	9, 10
	$\lambda$	0.1,5	0.1, 5	0.1, 5	0.1, 5
4	$\delta X_{\infty }$	− 21, 10	− 23, 10	8, 10	7, 10
	$\delta X_{0}$	− 23, 10	− 21, 10	9, 10	9, 10
	$\lambda$	0.1, 5	0.1, 5	0.1, 5	0.1, 5
1	$\Delta \overline{X}_{d - t}$	− 4, 5	− 3, 5	0.5, 5	1.5, 5
2	$\Delta \overline{X}_{d - t}$	3, 5	5, 5	0.1, 5	0.9, 5
3	$\Delta \overline{X}_{d - t}$	3, 5	4, 5	7, 5	6, 5
4	$\Delta \overline{X}_{d - t}$	5.5, 5	4.5, 5	8, 5	6, 5

Diet-tissue discrimination factors (DTDFs) were calculated as follows:

$$\Delta X_{diet - tissue} = \delta X_{eq.tissue} - \delta X_{diet} ,$$

where $\Delta X_{diet - tissue}$ is the DTDF value, $\delta X_{eq.tissue}$ referred to as the equilibrium tissue, which represented observed experimental tissue isotopic values that corresponded to the $\delta X_{\infty }$ from the isotope incorporation models, and $\delta X_{diet}$ is the isotopic values for the experimental diet sources that are given in Table 1. The Bayesian posterior distribution estimates for the DTDF, which were based on Markov Chain Monte Carlo (MCMC) simulation, were expressed as:

$$p(\Delta \overline{X}_{d - t} ,\sigma_{\Delta X}^{2} |\Delta X_{d - t} ) \propto \prod\limits_{i = 1}^{n} \mathcal{L} (\Delta X_{d - t} |\Delta \overline{X}_{d - t} ,\sigma_{\Delta X}^{2} ) \times \pi (\sigma_{\Delta X}^{2} )\pi (\Delta X_{d - t} ),$$

where $\Delta \overline{X}_{d - t}$ is the average DTDF and $\Delta X_{d - t}$ is the observed DTDF values for tissues that were considered to be at equilibrium. Prior distribution of $\Delta \overline{X}_{d - t}$ was specified based on normal distribution. Informative priors were based on the observed DTDF values from the experiment (Table 2).

### Isotope mixing model for experimental data

Sample-size corrected standard ellipse area (SEA_c_)^58^ was used to discern whether there were distinguishable differences among the five consumer groups of fish that were fed sources with different carbon and nitrogen stable isotope values. The bivariate Bayesian-based mixing model, *MixSIAR*^51^ was used to compare the effect of different DTDFs on the inference of the dietary composition of different fish consumer groups. The data for the different consumer groups included measurements from day 60 onwards since most of these corresponded to the $\delta X_{eq.tissue}$ values. Three DTDFs categories were used in the analyses, and these included literature-derived values ($\Delta {}^{13}C$ = 1.0 $\pm$ 2.0‰ and $\Delta {}^{15}N$ = 3.4 $\pm$ 2.0‰), and both the average $\Delta X_{diet - tissue}$ and the Bayesian inferred DTDFs that were derived from this experiment.

Similar to the isotope incorporation models, the isotope mixing models’ MCMC simulations and their posterior distributions were based on three Geweke chains with 100,000 iterations per chain, 50,000 burn-ins and a thinning interval of 3. For both the Bayesian isotope incorporation and the mixing models analyses, the MCMC model convergences were evaluated using the Gelman-Rubin diagnostic statistics, with the potential scale reduction factor (PSRF) values of approximately 1 considered as appropriate for model convergence^59^.

## Results

### Isotope incorporation parameter estimates and diet-tissue discrimination factors

**Figure 2:**
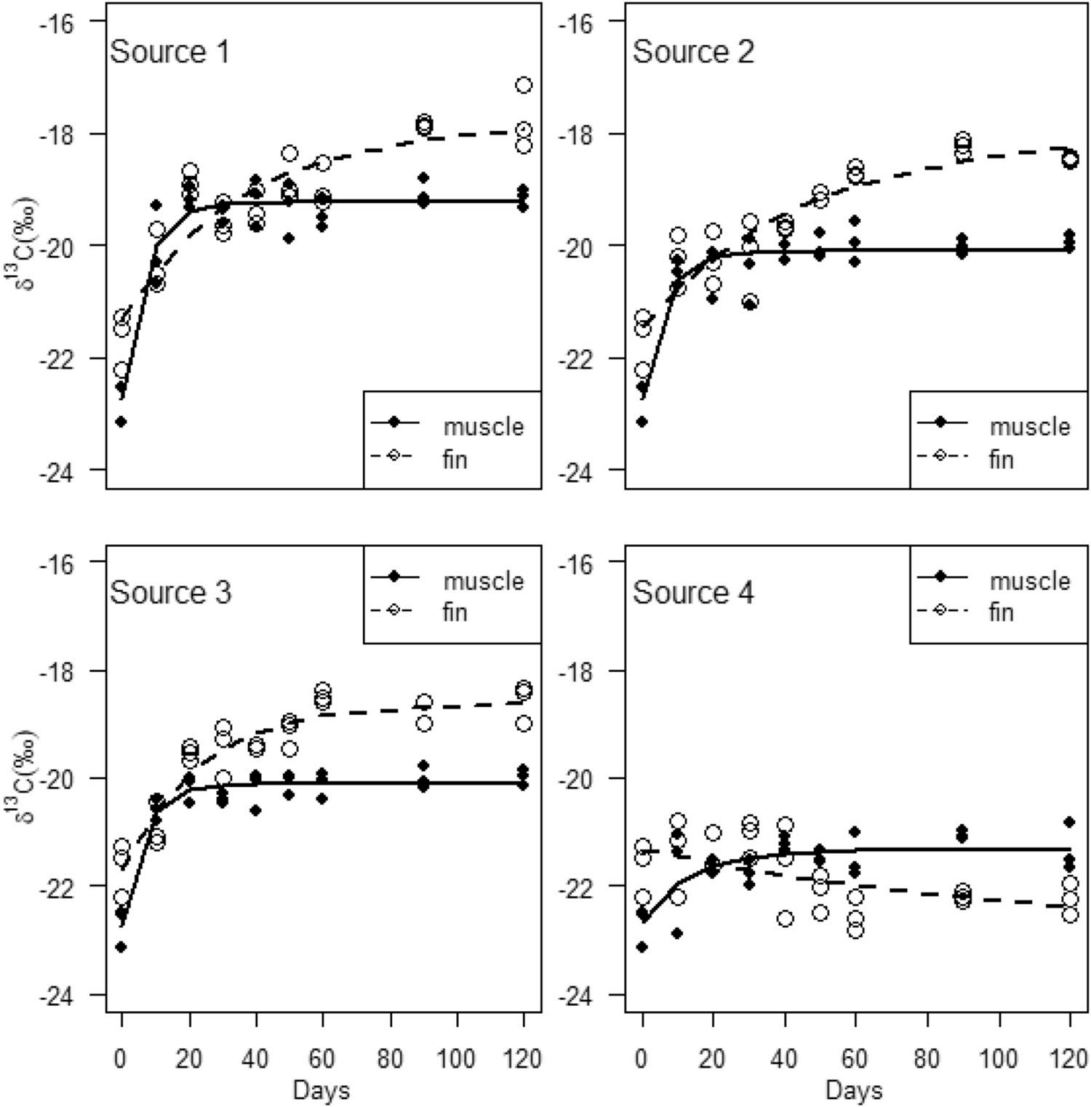
Isotopic incorporation of $\delta^{13} C$ into muscle and fin tissues of *Oreochromis mossambicus*. The curves illustrate model fit based on one-compartment models.

The $\delta^{13} C$ incorporation into muscle tissue exhibited temporal asymptotically increases for all sources (Fig. 2). Based on the DIC values, isotopic incorporation was best explained by both one-compartment (sources 1 and 4) and two-compartment (sources 2 and 3) models (Table 3). The $\delta^{13} C_{\infty }$ parameter estimates varied among the different groups, with a high value in consumer group 1 ($\delta^{13} C_{\infty }$ = − 19.23‰, CR = − 19.4 to − 19.1‰) followed by consumer groups 2 ($\delta^{13} C_{\infty }$ = − 20.06‰, CR = − 20.2 to − 19.7‰) and 3 ($\delta^{13} C_{\infty }$ = − 20.09‰, CR = − 20.2 to − 19.8‰), and a low value in consumer group 4 ($\delta^{13} C_{\infty }$ = − 21.36‰, CR = − 21.6 to − 21.1‰) (Table 3). The average residence times were also variable, and ranged from approximately 2 to 5 days. For fin tissue, $\delta^{13} C$ incorporation showed temporal asymptotic increases in consumer groups 1, 2 and 3, but exhibited a temporal decrease in consumer group 4 (Fig. 2). Both one-compartment (sources 3 and 4) and two-compartment (sources 1 and 2) models were important in explaining $\delta^{13} C$ incorporation into fin tissue (Table 3). The fin tissue $\delta^{13} C_{\infty }$ parameter estimates were higher in consumer groups 1 ($\delta^{13} C_{\infty }$ = − 17.86‰, CR = − 18.3 to − 17.3‰), 2 ($\delta^{13} C_{\infty }$ = − 18.48‰, CR = − 18.9 to − 18.1‰) and 3 ($\delta^{13} C_{\infty }$ = − 18.51‰, CR = − 18.8 to − 18.2‰) and lower in consumer group 4 ($\delta^{13} C_{\infty }$ = − 22.88‰, CR = − 23.5 to − 22.2‰) compared to those of the muscle tissue. For the best-supported models, the average residence times of $\delta^{13} C$ incorporation into fin tissue was relatively higher, ranging from 33 to 100 days compared to those of muscle tissue for all the diets groups. This indicated that muscle tissue has relatively faster isotope turnover rates compared to fin tissue.

**Table 3:** Posterior estimates for the parameters of one- and two-compartment models. The values indicate the means and the Bayesian 95% credibility ranges (CR) in parentheses.

Isotope/source	Tissue	One compartment				Two compartment					
		$\delta X_{0}$	$\delta X_{\infty }$	$\lambda$	*DIC*	$\delta X_{0}$	$\delta X_{\infty }$	$\lambda_{1}$	$\lambda_{2}$	$p$	*DIC*
$\delta^{13} C$ 1	Muscle	− 22.83 (− 23.2, − 22.5)	− 19.23 (− 19.4, − 19.1)	0.20 (0.12, 0.69)	28.03	− 23.83 (− 23.2, − 22.5)	− 19.22 (− 19.4, − 19.0)	0.38 (0.05, 1.26)	0.39 (0.05, 1.27)	0.50 (0.03, 0.97)	28.46
	Fin	− 21.20 (− 21.5, − 20.7)	− 17.95 (− 18.4, − 17.4)	0.03 (0.02, 0.05)	48.66	− 21.34 (− 21.8, − 20.9)	− 17.86 (− 18.3, − 17.3)	0.33 (0.01, 1.32)	0.30 (0.01, 1.25)	0.49 (0.04, 0.96)	46.02
2	Muscle	− 22.81 (− 23.2, − 22.5)	− 20.07 (− 20.2, − 19.9)	0.37 (0.10, 1.23)	25.72	− 22.51 (− 22.8, − 22.2)	− 20.06 (− 20.2, − 19.7)	0.45 (0.02, 1.33)	0.46 (0.02, 1.34)	0.50 (0.05, 0.95)	23.50
	Fin	− 21.35 (− 21.7, − 21.0)	− 18.48 (− 18.9, − 18.1)	0.03 (0.02, 0.04)	39.40	− 21.42 (− 21.8, − 21.0)	− 18.48 (− 18.9, − 18.0)	0.36 (0.02, 1.32)	0.28 (0.02, 1.22)	0.47 (0.01, 0.98)	39.84
3	Muscle	− 22.78 (− 23.1, − 22.5)	− 20.14 (− 20.3, − 20.0)	0.27 (0.13, 1.03)	6.62	− 22.78 (− 23.0, − 22.5)	− 20.09 (− 20.2, − 19.8)	0.41 (0.02, 1.30)	0.41 (0.01, 1.30)	0.50 (0.04, 0.96)	5.34
	Fin	− 21.78 (− 22.1, − 21.4)	− 18.51 (− 18.8, − 18.2)	0.04 (0.03, 0.06)	28.61	− 21.82 (− 22.2, − 21.5)	− 18.40 (− 18.8, − 17.8)	0.24 (0.01, 1.18)	0.28 (0.01, 1.26)	0.51 (0.01, 0.99)	30.31
4	Muscle	− 22.82 (− 23.2, − 22.4)	− 21.36 (− 21.6, − 21.1)	0.23 (0.04, 0.92)	33.95	− 22.45 (− 22.8, − 22.0)	− 21.32 (− 21.6, − 20.8)	0.47 (0.01, 1.36)	0.46 (0.01, 1.35)	0.50 (0.03, 0.97)	34.55
	Fin	− 21.52 (− 21.9, − 21.2)	− 22.88 (− 23.5, − 22.2)	0.01 (0.00, 0.02)	47.36	− 21.15 (− 21.5, − 20.7)	− 22.82 (− 23.5, − 22.2)	0.26 (0.00, 1.25)	0.29 (0.00, 1.26)	0.52 (0.01, 0.99)	48.70
$\delta^{15} N$ 1	Muscle	8.71 (8.4, 9.0)	10.41 (10.2, 10.7)	0.05 (0.03, 0.09)	22.06	8.67 (8.3, 9.0)	10.40 (10.1, 10.7)	0.30 (0.02, 1.24)	0.30 (0.02, 1.23)	0.50 (0.01, 0.98)	22.24
	Fin	8.72 (8.3, 9.1)	11.42 (11.1, 11.7)	0.05 (0.03, 0.07)	37.32	8.68 (8.2, 9.0)	11.41 (11.1, 11.7)	0.27 (0.03, 1.21)	0.27 (0.03, 1.21)	0.50 (0.01, 0.99)	39.67
2	Muscle	8.67 (8.3, 9.0)	10.65 (10.4, 10.9)	0.07 (0.04, 0.12)	26.67	8.65 (8.2, 9.0)	10.65 (10.4, 10.9)	0.29 (0.03, 1.23)	0.30 (0.03, 1.23)	0.50 (0.01, 0.99)	28.90
	Fin	8.80 (8.3, 9.2)	11.48 (11.2, 11.8)	0.06 (0.04, 0.09)	36.51	8.76 (8.3, 9.2)	11.48 (11.2, 11.8)	0.29 (0.03, 1.23)	0.29 (0.03, 1.23)	0.50 (0.01, 0.98)	38.62
3	Muscle	9.04 (8.8, 9.3)	8.95 (8.8, 9.3)	0.59 (0.02, 1.39)	9.64	9.04 (8.8, 9.3)	8.95 (8.8, 9.1)	0.59 (0.00, 1.39)	0.59 (0.00, 1.39)	0.48 (0.02, 0.97)	9.68
	Fin	9.35 (9.1, 9.6)	7.12 (6.7, 7.5)	0.02 (0.01, 0.03)	10.73	9.38 (9.1, 9.6)	7.08 (6.6, 7.4)	0.27 (0.01, 1.22)	0.25 (0.01, 1.18)	0.49 (0.01, 0.99)	11.76
4	Muscle	9.04 (8.7, 9.4)	8.51 (8.3, 8.6)	0.52 (0.05, 1.34)	24.64	9.04 (8.7, 9.4)	8.49 (8.2, 8.7)	0.55 (0.01, 1.37)	0.55 (0.01, 1.38)	0.50 (0.03, 0.97)	24.55
	Fin	9.17 (8.8, 9.5)	6.38 (5.7, 6.9)	0.02 (0.01, 0.03)	24.72	9.29 (9,0 9.6)	6.25 (5.6, 6.8)	0.24 (0.00, 1.19)	0.28 (0.00, 1.16)	0.52 (0.02, 0.98)	24.12

Comparisons between one- and two-compartment models were done using the deviance information criterion (DIC).

**Figure 3:**
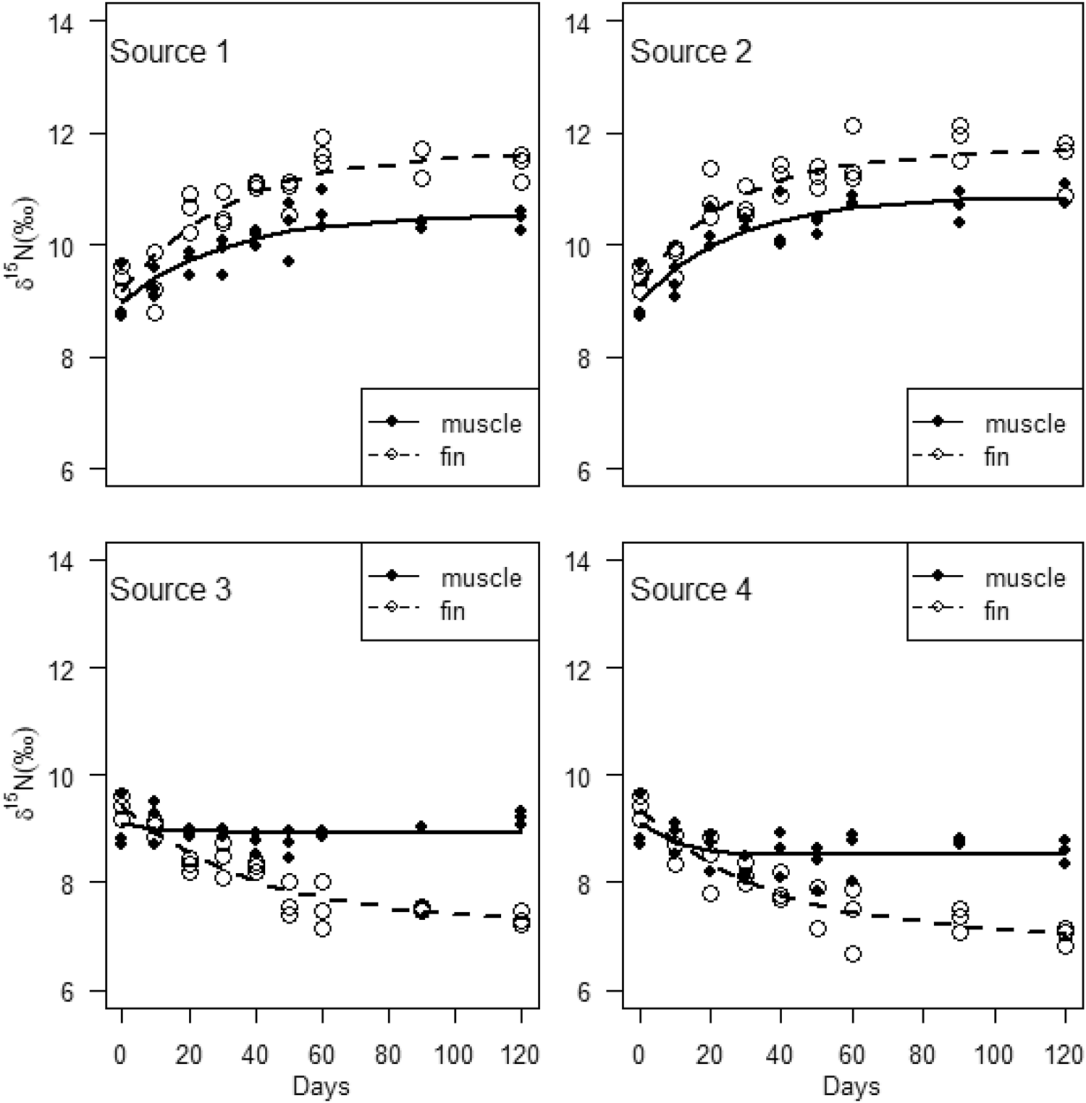
Isotopic incorporation of $\delta {}^{15}N$ into muscle and fin tissues of *Oreochromis mossambicus*. The curves illustrate model fit based on one-compartment models.

The $$\delta {}^{15}N$$ incorporation patterns into muscle and fin tissues were best supported by one-compartment models in all consumer groups, except in consumer group 4 that was best supported by two-compartment models (Table 3). For muscle tissue, $$\delta^{15} N$$ incorporation increased asymptotically over time in consumer groups 1 and 2, whereas consumer groups 3 and 4 showed a relatively slight decrease (Fig. 3). The $$\delta^{15} N_{\infty }$$ parameter estimates were higher in consumer groups 1 ($$\delta^{15} N_{\infty }$$ = 10.41‰, CR = 10.2 to 10.7‰) and 2 ($$\delta^{15} N_{\infty }$$ = 10.65‰, CR = 10.4 to 10.9‰) compared to consumer groups 3 ($$\delta^{15} N_{\infty }$$ = 8.95‰, CR = 8.8 to 9.3‰) and 4 ($$\delta^{15} N_{\infty }$$ = 8.49‰, CR = 8.2 to 8.7‰). Consequently, the average residence times were higher for consumer groups 1 ($$\tau$$$$\approx$$ 20 days) and 2 ($$\tau$$$$\approx$$ 14 days) than for consumer groups 3 ($$\tau$$$$\approx$$ 2 days) and 4 ($$\tau$$$$\approx$$ 2 days). For the fin tissue, the $$\delta {}^{15}N$$ incorporation was characterised by asymptotic increase in consumer groups 1 and 2, and pronounced asymptotic decrease in consumer groups 3 and 4 (Fig. 3). The best-supported models indicated high $$\delta^{15} N_{\infty }$$ values in consumer groups 1 ($$\delta^{15} N_{\infty }$$ = 11.42‰, CR = 11.1 to 11.7‰) and 2 ($$\delta^{15} N_{\infty }$$ = 11.48‰, CR = 11.2 to 11.8‰) and low values in consumer groups 3 ($$\delta^{15} N_{\infty }$$ = 7.12‰, CR = 6.7 to 7.5‰) and 4 ($$\delta^{15} N_{\infty }$$ = 6.38‰, CR = 5.7 to 6.9). The average residence times were variable among the different consumer groups, ranging from approximately 17 to 50 days.

The average DTDFs and Bayesian based MCMC-DTDFs were comparable for all the consumer groups, but varied among the different consumer groups and between different body tissues (Table 4). For muscle tissue, $$\Delta {}^{13}C$$ was most negative for consumer group 1 ($$\Delta {}^{13}C$$ = − 4.4), which was fed isotopically-enriched diet source, and most positive for consumer group 4 ($$\Delta {}^{13}C$$ = 5.6), which was fed isotopically-depleted diet source, compared to consumer groups 2 and 3 that were fed diet sources with intermediate $$\delta^{13} C$$ values (Table 4). For fin tissue, $$\Delta {}^{13}C$$ were higher for consumer groups 2 ($$\Delta {}^{13}C$$ = 5.2), 3 ($$\Delta {}^{13}C$$ = 4.1) and 4 ($$\Delta {}^{13}C$$ = 4.6) than for consumer group 1 ($$\Delta {}^{13}C$$ = − 3.1). Similar to $$\Delta {}^{13}C$$, the muscle tissue $$\Delta {}^{15}N$$ values were highest for consumer groups that were fed diet sources with low isotope values ($$\Delta {}^{15}N$$$$\approx$$ 8 for both consumer groups 3 and 4) compared to consumer groups that were fed diet sources with high isotope values ($$\Delta {}^{15}N$$ < 1 for both consumer groups 1 and 2) (Table 4). A consistent pattern was observed for fin tissue $$\Delta {}^{15}N$$, whereby consumer groups that were fed diet sources with high stable isotope values had low DTDFs compared to consumer groups that were fed diet sources with low stable isotope values.

**Table 4:** Diet-specific discrimination factors for muscle and fin tissues that were derived from feeding experiment for *Oreochromis mossambicus*.

Diet	Tissue	Mean		MCMC	
		$\Delta {}^{13}C$	$\Delta {}^{15}N$	$\Delta {}^{13}C$	$\Delta {}^{15}N$
1	Muscle	− 4.4 ± 0.2	0.5 ± 0.2	− 4.4 (− 4.7, − 4.2)	0.5 (0.3, 0.6)
	Fin	− 3.1 ± 0.4	1.5 ± 0.3	− 3.2 (− 3.6, − 2.7)	1.5 (1.2, 1.9)
2	Muscle	3.5 ± 0.1	0.2 ± 0.2	3.5 (3.4, 3.7)	0.1 (− 0.3, 0.4)
	Fin	5.2 ± 0.1	0.7 ± 0.5	5.2 (5.0, 5.4)	0.9 (0.4, 1.5)
3	Muscle	2.9 ± 0.2	7.8 ± 0.1	2.9 (2.7, 3.1)	7.7 (7.5, 7.9)
	Fin	4.1 ± 0.3	5.9 ± 0.1	4.1 (3.8, 4.5)	6.0 (5.8, 6.2)
4	Muscle	5.6 ± 0.3	7.8 ± 0.2	5.6 (5.1, 6.0)	7.9 (7.7, 8.1)
	Fin	4.6 ± 0.2	6.2 ± 0.2	4.6 (4.3, 4.8)	6.4 (6.1, 6.7)

The values were obtained using average (mean ± standard deviation) DTDFs based on differences between source and equilibrium tissue isotopic values, and Bayesian-based Markov chain Monte Carlo (MCMC) simulations with values presented as means and credibility intervals in parentheses.

### Isotope mixing models

**Figure 4:**
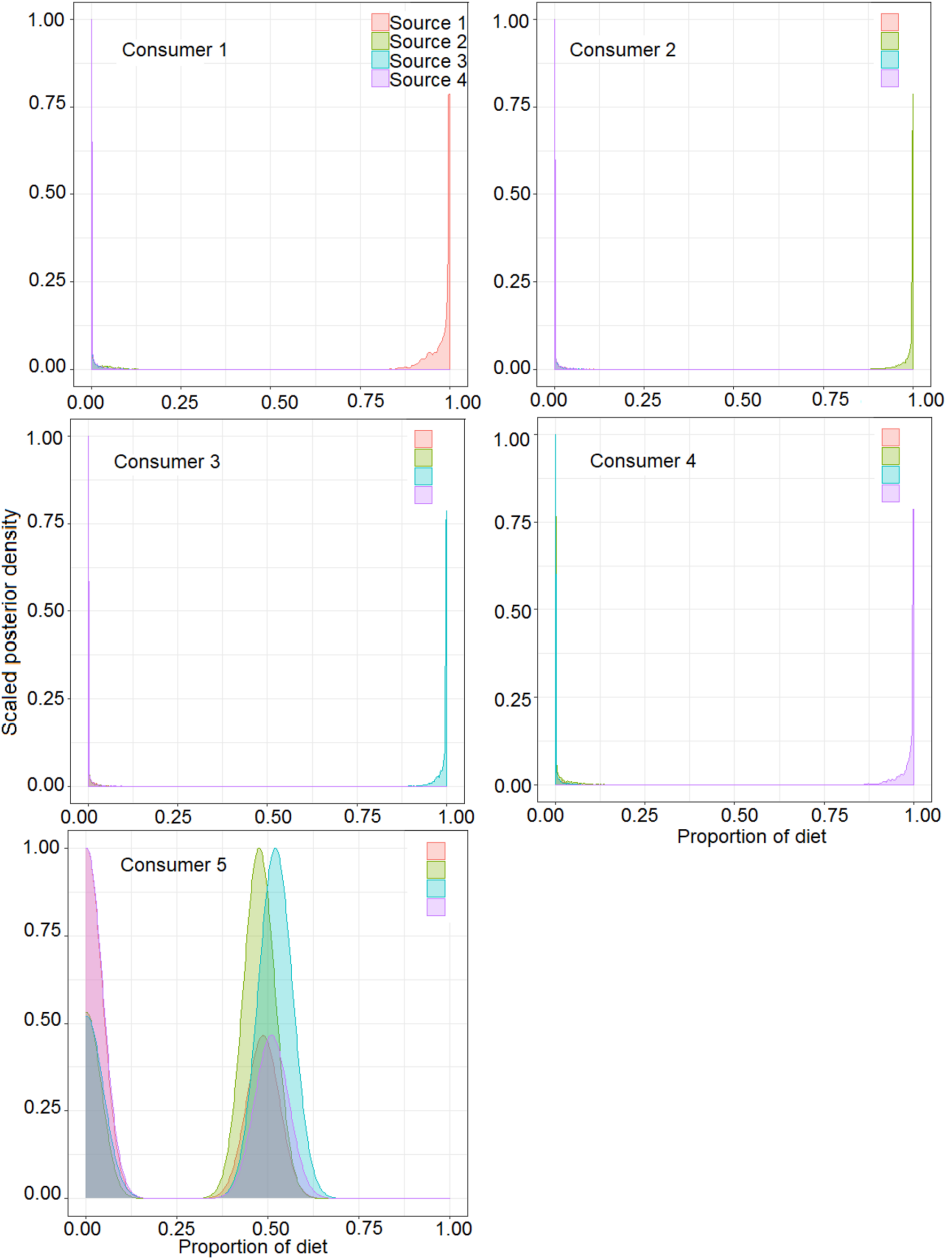
MixSIAR estimated source contributions for simulated consumer groups showing Bayesian credibility intervals and posterior densities.

For the hypothetical data, the *MixSIAR* model showed that the single-source consumer groups predominantly reflected their respective diet sources (mean > 98%, 95% credibility range (CR), approximately 90–100% for all sources) (Fig. 4). The multiple-source consumer group showed higher proportional contributions of sources 2 (mean = 31.7%, 95% CR = 0.0–50.2%) and 3 (mean = 34.8%, 95% CR = 0.0–54.8%) than sources 1 (mean = 16.4%, 95% CR = 0.0–51.2%) and 4 (mean = 17.1%, 95% CR = 0.0–53.5%). The high CR values indicated that for a well-mixed consumer group, the estimations of the proportional contributions of multiple diet sources were characterised by high uncertainty.

**Figure 5:**
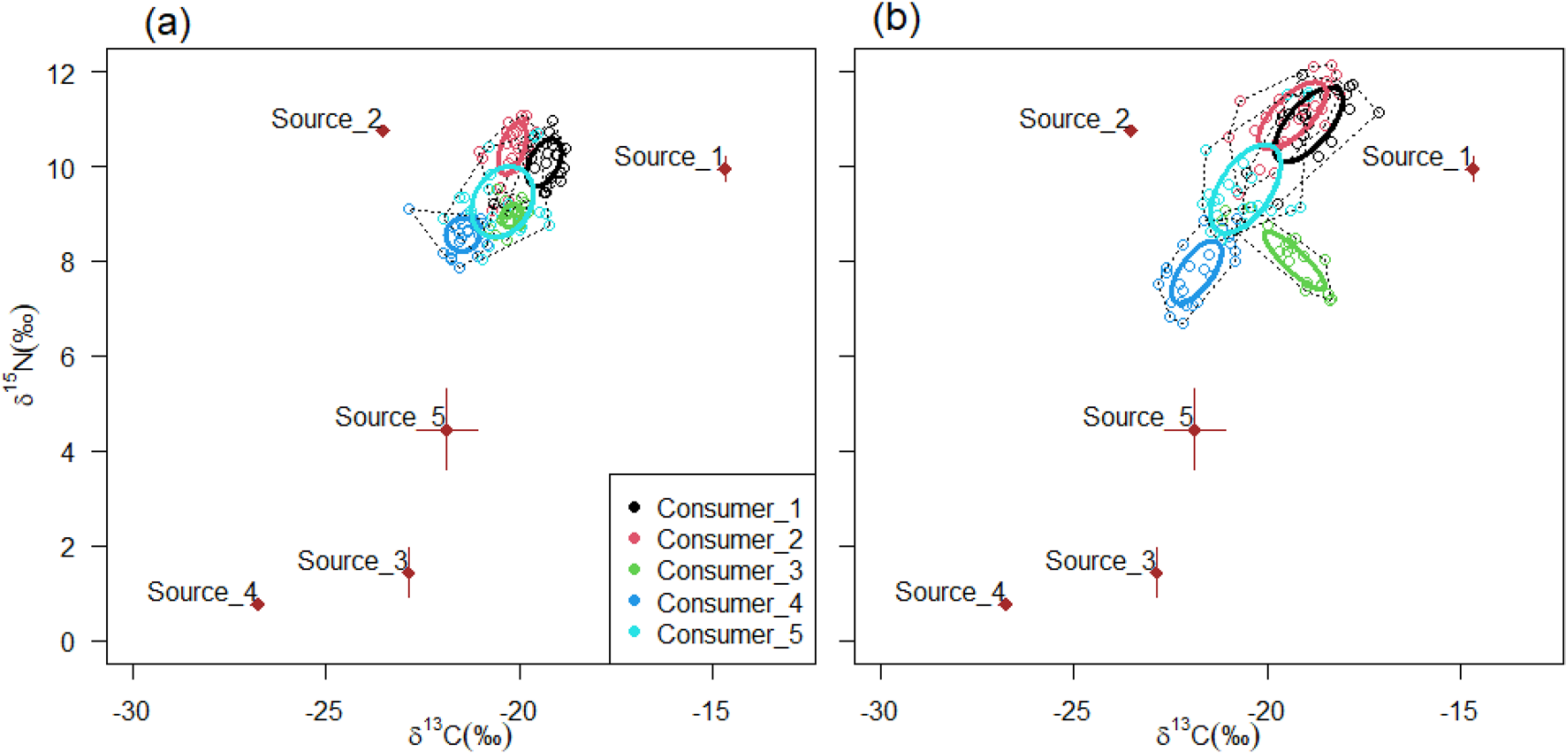
Inter- and intra-group variation, and sample size corrected standard ellipse area (SEA_c_) based on muscle (**a**) and fin (**b**) tissues for *Oreochromis mossambicus* that were fed diets with different $\delta^{13} C$ and $\delta {}^{15}N$ values. The values for the diet sources include the mean and standard deviations for $\delta^{13} C$ and $\delta {}^{15}N$. The consumer groups comprise convex hulls, which encompass all individuals, and SEA_c,_ which encompass 40% of the sample.

**Figure 6:**
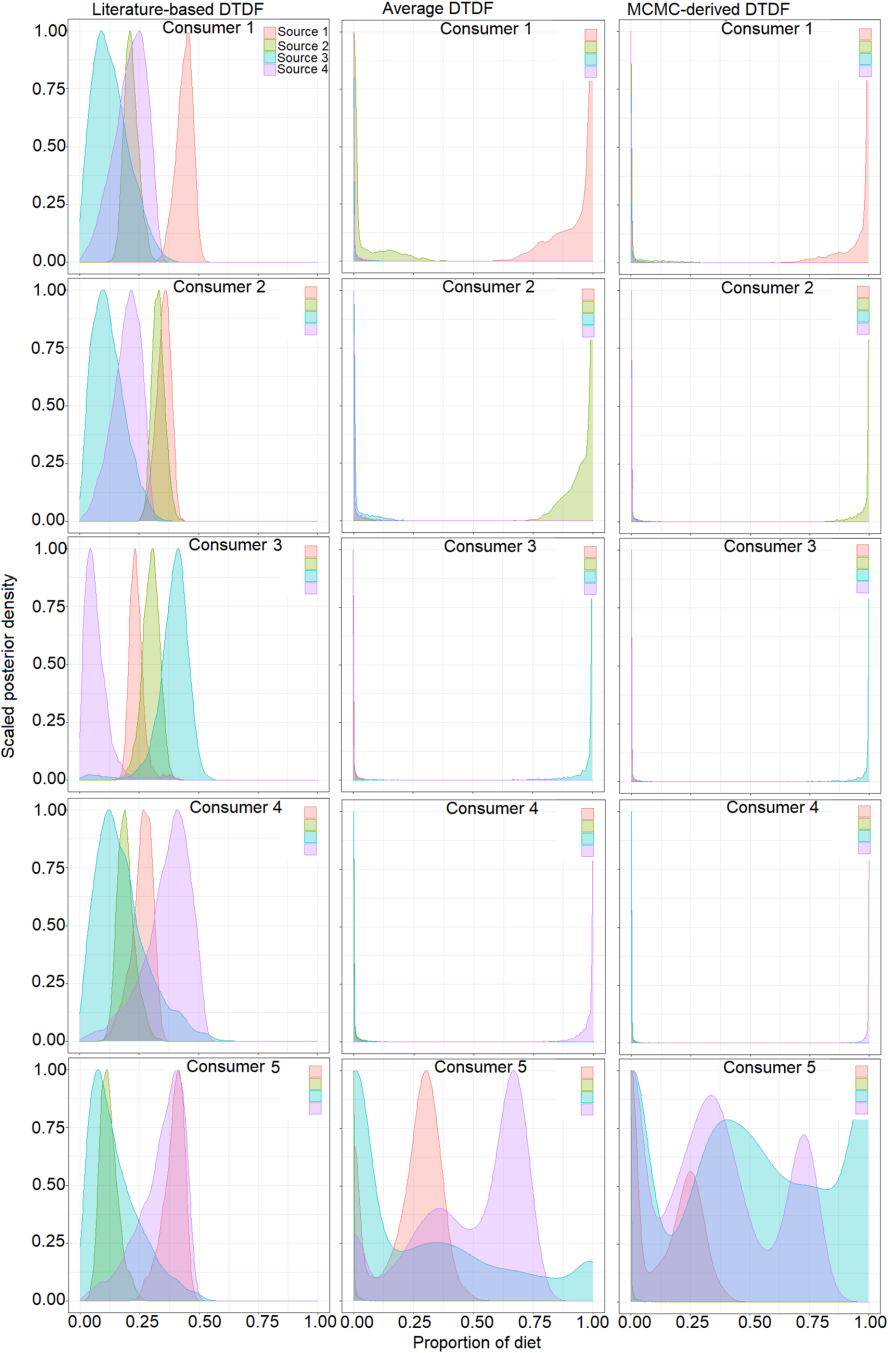
Mixing model estimated dietary contributions inferred for muscle tissue based on MixSIAR using either literature-derived or diet-specific discrimination factors. Density plots show Bayesian credibility intervals for each diet source.

For the *O. mossambicus* experimental trial, muscle data indicated that the single-source consumer groups (consumers 1 to 4) were distinguishable by non-overlapping SEA_c_ (Fig. 5a). In addition, the mixed-source group (consumer 5) was distinguishable because it was generally intermediate to the single-source groups. The application of literature-derived discrimination factors showed that all the single-source consumer groups’ respective diet sources contributed < 50% (consumer 1, source 1 mean = 44.0%, 95% CR = 36.8–46.8%; consumer 2, source 2 mean = 33.1%, 95% CR = 28.2–38.7%; consumer 3, source 3 mean = 39.1%, 95% CR = 21.0–49.2%; and consumer 4, source 4 mean = 36.7%, 95% CR = 13.1–50.5%) (Fig. 6). The application of diet-specific average DTDFs and MCMC-based DTDFs mixing models for muscle tissue yielded comparable dietary estimates. In contrast to literature-derived DTDFs estimates, the average DTDFs and the MCMC-derived DTDFs mixing model estimates revealed high proportional contributions for each of the diet sources for the respective single-source consumer groups (Fig. 6). The mean proportional contribution of the respective diet sources ranged between approximately 94–99%. For the mixed-source consumer group (consumer 5), the application of literature-based DTDFs revealed sources 1 (mean = 33.8%, 95% CR = 9.3–45.9%) and 4 (mean = 29.9%, 95% CR = 22.0–36.3%) as most important compared to sources 2 and 3. By comparison, the average DTDF mixing model estimates showed high proportional contributions of sources 1 (mean ≈ 27.8%, CR ≈ 0.0–56.0%) and 4 (mean ≈ 47.9%, CR ≈ 0.0–90.0%). The MCMC-derived DTDF mixing model estimates were characterised by high uncertainty, with three sources (source 1, 3 and 4) being inferred as important (Fig. 6).

**Figure 7:**
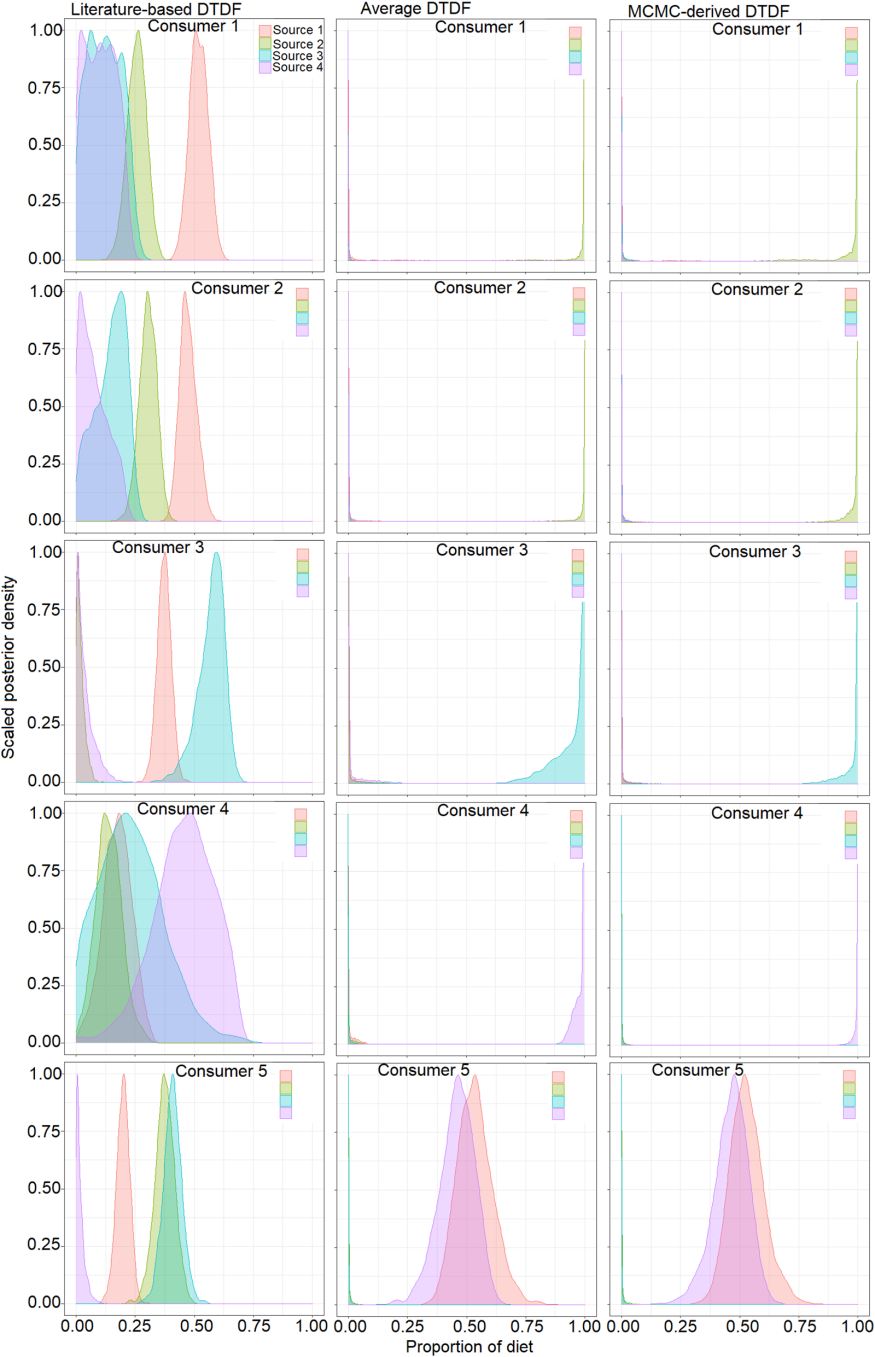
Mixing model estimated dietary contributions inferred for fin tissue based on simmr using either literature-derived or diet-specific discrimination factors. Density plots show Bayesian credibility intervals for each diet source.

Fin tissue consumer groups were characterised by large intragroup variations together with a high overlap in the SEA_c_ between consumer groups 1 and 2 (Fig. 5b). The application of literature-derived DTDFs mixing models in the single-source groups showed that the contributions of respective diet sources were low and variable (consumer 1, source 1 mean = 51.9%, 95% CR = 44.5–59.3%; consumer 2, source 2 mean = 30.3%, 95% CR = 27.7—37.1%; consumer 3, source 3 mean = 57.1%, 95% CR = 43.5–66.0%; and consumer 4, source 4 mean = 45.4%, 95% CR = 12.7–66.5%) (Fig. 7). In comparison, application of the average DTDFs and the MCMC-derived DTDFs for single-source consumer groups revealed that the proportional contributions of their respective diet sources was > 95% (Fig. 7). An exception was consumer group 1 where diet source 2 was inferred as most important. For the mixed-source consumer group, the literature-derived DTDFs mixing model estimates showed higher contributions for sources 2 (mean = 37.5%, 95% CR = 28.7–45.5%) and 3 (mean = 40.8%, 95% CR = 32.9–48.8%) than sources 1 (mean = 19.9%, 95% CR = 14.1–25.2%) and 4 (mean = 2.0%, 95% CR = 0.0–7.1%). By comparison, the diet-specific DTDF-based mixing models revealed relatively high contributions by diet sources 1 and 4 (Fig. 7).

## Discussion

### Patterns in isotope incorporation and DTDFs

Consistent with our study’s first hypothesis, which predicted diet source and tissue differences, there were considerable variations in both the isotope incorporation patterns and the DTDFs for the muscle and fin tissues of *O. mossambicus* groups that were fed different diet sources. Specifically, for $\delta^{13} C$, all consumer groups showed a progressive temporal increase in the carbon stable isotope values, except consumer group 4. In addition, variation in $\delta^{13} C_{\infty }$ estimates corresponded with that of the source $\delta^{13} C$ values. On the other hand, the DTDFs were negative for both muscle and fin tissues of consumer group 1, whereas for the rest of the consumer groups, the DTDFs were positive, ranging from approximately 3 to 6. By comparison, for $\delta^{15} N$, consumer groups 1 and 2, which were fed $\delta^{15} N$-enriched sources, showed progressive temporal increase in the stable isotope values of their tissues, whereas consumer groups 3 and 4, which were fed $\delta^{15} N$-depleted sources, showed progressive decrease in the stable isotope values of their tissues. Conversely, consumer groups 1 and 2 were distinguished by having low nitrogen DTDFs ($\Delta^{15} N$ < 2), whereas consumer groups 3 and 4 were characterised by high nitrogen DTDFs ($\Delta^{15} N$ > 6) for all tissues. Overall, these observations indicated variability in isotopic incorporation patterns and diet to tissue differences, which provides empirical evidence on the likely influence of diet composition and tissue type on variation in both isotope incorporation patterns and DTDFs. This pattern appeared consistent with those observed in several studies on both fishes^36,37,49,60^ and other animal taxa^27,32,33^.

In several general studies on different animal taxa, the influence of diet composition on both isotopic incorporation and DTDFs of consumers has been widely associated with the nature of dietary carbohydrates and proteins, together with their metabolism. For the $\delta^{13} C$, dietary carbohydrate sources are often distinguished based on their primary photosynthetic carbon fixing pathway, particularly between C_3_ and C_4_ pathways, which yield high and low $\delta^{13} C$ values, respectively, in consumed diets^61^. This was particularly so in our study where the dietary carbon sources were distinguished based on either high proportion of C_3_-derived carbohydrates, which had depleted $\delta^{13} C$ values, or high proportion of C_4_-derived carbohydrates, which were enriched in $\delta^{13} C$. These dietary differences in stable isotope values appeared to correspond with the tissue isotopic differences among the experimental animal groups. Specifically, larger $\Delta^{13} C$ were observed for consumer groups that were fed sources that were either depleted or highly enriched in $\delta^{13} C$ compared to consumer groups that were fed on diet sources with intermediate $\delta^{13} C$ values. Several studies elsewhere have reported that metabolism of the food sources with inherent differences in $\delta^{13} C$ undergo different kinetic pathways in isotopic assimilation, which ultimately influences both isotope incorporation patterns and the DTDFs in consumer tissues^16,17,25^. For example, DeNiro and Epstein^17^ showed that trophic enrichment of $\delta^{13} C$ varied in relation to source $\delta^{13} C$ values because the metabolic biochemical fractions would directly depend on both the quantity and quality of dietary isotope values. Recent empirical studies on fishes have also shown that different diet sources can yield considerable variation in both tissue turnover rates and $\Delta^{13} C$^36,42,62,63^. Similarly, an extensive review by Caut et al.^29^ on several animal taxa reported wide ranging $\Delta^{13} C$ values (− 8.8 to 6.1‰) due to several factors, including diet kinetic effects during catabolism and macromolecule synthesis.

Similar to $\delta^{13} C$, our study showed different $\delta^{15} N$ incorporation patterns and the associated DTDFs among the consumer groups. Specifically, there were comparable patterns in consumer groups that were fed animal protein-based diets (sources 1 and 2), with these groups exhibiting both high tissue enrichment and low $\Delta^{15} N$ values. Conversely, consumer groups that were fed plant protein-based diets (sources 3 and 4) were distinguished by having high $\Delta^{15} N$ values and corresponding low $\delta^{15} N$ muscle and fin tissue enrichment patterns. These observations suggest the importance of protein quality, which is regarded as central in influencing nitrogen stable isotope incorporation patterns^21,64,65^. It has generally been reported that in addition to the inherent differences in $\delta^{15} N$ between animal- and plant-protein based diets^25^, animal protein diets tend to show low DTDFs and high assimilation rates, whereas plant protein diets are usually associated with high DTDFs^29,50^. This is because animal protein diets are assumed to readily satisfy the amino acid requirements of consumers, resulting in both the protein and lipid content of consumer tissues being closely matched to those of their diets^64,66^. In contrast, the amino acids from plant protein diets are often different, and usually undergo different metabolic pathways, such as the transamination of keto acids from the carbohydrates in order to satisfy the specific amino acid requirements for consumer animals^67^. This result in different assimilation and fractionation of $\delta^{15} N$ into tissues^20,67^. Mill et al.^35^ further reported that fishes that rely on plant dietary protein had to consume high daily rations in order to satisfy their bioenergetic requirements. Therefore, the high intake of $\delta^{15} N$-low food has been inferred to result in a corresponding increase in the diet-to-tissue isotopic differences^36^. The variation in the isotopic incorporation patterns from our study thus appeared to be consistent with this large body of literature that shows the different fates of dietary stable isotopes in animal tissues.

In addition to diet differences in isotope patterns, our study showed observable tissue differences in isotope incorporation and DTDFs. In particular, fin tissue was observed to be more variable and exhibited lower turnover rates together with high DTDFs compared to muscle tissue. These differences were further shown by variation in the support for one-compartment and two-compartment models in the isotope incorporation patterns for the different diet sources. Specifically, one-compartment models best supported $\delta^{13} C$ incorporation in muscle tissue for consumer groups that were fed sources with the highest (source 1) and the lowest (source 4) isotopic values. By comparison, one-compartment models best supported $\delta^{13} C$ incorporation in fin tissue for most consumer groups, except consumer group 1 that was fed a source with high isotopic values. Our results further showed that one-compartment models best supported $\delta^{15} N$ incorporation in both tissues all for most consumer groups, except consumer group 4 was fed a source with low isotopic values. Although the physiological mechanisms that underpin the support for multiple compartment models are less understood^55^, multiple compartment models have been shown to reflect longer residence times in isotopic turnover^37^. Our results, thus, highlight source and tissue differences on aspects such as isotopic turnover rates and incorporation patterns. Comparative studies on fishes have shown that tissue variations in isotopic patterns may be related to either differences in amino acid, lipid and carbohydrate requirements during macromolecule synthesis^68^ or to differences in the catabolic turnover between active and structural tissues^37,69,70^. In this study, the tissue differences suggest disparities in tissue metabolism, such as the addition of new tissue through growth versus catabolic processes, which have been observed in other studies (e.g.^71^). Thus, the general fast turnover in muscle compared to fin tissue suggests either differences in protein and lipid content^72^ or high muscle turnover compared to fin tissue. Although this observation appeared to be consistent with some studies^38,42^, other studies have, nonetheless, reported faster turnover and low isotope enrichment in fin tissues compared to muscle tissue^37,63,71^. These variations therefore highlight the importance of interspecific differences in isotope incorporation and the need to understand the aspects related to tissue metabolism.

### Isotope mixing model

A critical aspect when applying isotope mixing models is using DTDFs to address the systematic differences in isotope values between food sources and consumer tissues^3^. This aspect was illustrated by our simulated data, which provided a conceptual framing that depicted dietary estimates for hypothetical consumer groups based on the commonly used DTDFs. Ideally, these DTDFs can be considered as correction factors that are essential in linking sources to consumer isotope signatures in order to provide the correct estimates for their dietary composition^3,13^. In our study, the dietary estimates for the hypothetical single-source consumer groups best illustrated this aspect. By comparison, the hypothetical multiple-source consumer group’s dietary estimates were characterised by high uncertainty with two sources being most important. This highlights critical aspects that need to be considered in the general application of isotope mixing models. This appears to be an unavoidable feature of isotope mixing models, in that in particularly well-mixed systems the model may not be able determine which sources are being consumed. The most likely values of 25% come from a generalist prior probability distribution, which may not be suitable for the system, though these are caveated by wide CRs. Therefore, practical applications of isotope mixing models require useful prior information on diet sources, an aspect that has been highlighted in previous studies (e.g.^3^).

Our experimental study using *O. mossambicus* revealed that applying the commonly used DTDFs, particularly for single-source consumer groups, underestimated the proportional contributions of the individual sources. On the other hand, and consistent with this study’s second hypothesis, the use of experiment-derived DTDFs provided insights that indicated the following aspects. First, the experiment-derived DTDFs provided evidence of better dietary estimates for the single-source consumer groups. Second, for the mixed-source consumer group, dietary estimates were variable, with the mixing model showing the importance of mostly two sources. This suggests the importance of understanding the foundational trophic ecology of the consumer, and some probable underlying metabolic processes, such as the likelihood of preferential metabolism of certain diets. Although *O. mossambicus* is generally omnivorous, it is known to exhibit an ontogenetic shift from carnivorous to predominantly herbivorous diet^73^. In this study, relatively higher dietary estimates were observed for diet source 1, which comprised maize and fish meal, and diet source 4, which comprised soya and rice as protein and carbohydrate sources, respectively.

The application of isotope mixing models on both the hypothetical and experimental data suggest that there are some important caveats that need critical consideration in estimating animal diets. Firstly, the above observations suggest that the use of either uninformative or incorrect DTDFs poses the risk of potential erroneous estimates of dietary composition while highlighting the sensitivity of these factors in isotope mixing models. Recent studies that have critiqued the use of isotope mixing models have indicated that erroneous application of DTDFs may be borne either due to inaccurate assumptions of the biological processes that underlie isotope tracers coupling into consumer tissues^8^ or due to failure to take into account the variation in the isotope values of different sources^74^. In our study, it appears that these shortcomings were underscored by both variations in isotope incorporation patterns and in diet-to-tissue differences among *O. mossambicus* consumer groups that were fed different sources, which adds to evidence from other studies on both fishes^36,49^ and other animal taxa^27,31,33^. Secondly, information on the underlying biological processes, particularly on the fate of different stable isotopes in different tissues, is essential when applying isotope mixing models.

The use of appropriate DTDFs in isotope mixing models has, nonetheless, become a subject of increasing attention in recent studies. For example, some studies that have explored both the biological processes and the fate of assimilated isotope tracers, and have applied experimentally derived factors in order to provide reasonable dietary estimates^38,75^. Despite the relevance of experimental studies in providing the appropriate DTDFs that can be used in isotope mixing models, and their recent application in studies involving both fishes^38,49,63^ and other animal taxa^27,76^, gathering this information still remains elusive for many taxa, in part due to experimental costs and interspecific differences that potentially confound generalisations. Nevertheless, alternative approaches such as using mathematical corrections have also been suggested. For example, recent field-based studies have applied different DTDFs through arithmetic corrections to distinguish between diet types^34^, whereas others have applied linear models to discern consumer and prey differences in DTDFs^40^. Similarly, in recent field research, Bastos et al.^41^ explored the use of different DTDFs in mixing models for an omnivorous fish, *Jenynsia multidentata*, that relied on herbivorous and carnivorous diets within coastal habitats in southern Brazil, whereas Hopkins et al.^4^ used variable correction factors for DTDFs to account for diet differences when they estimated the diets of grizzly bears, *Ursus arctos*, from the Greater Yellowstone Ecosystem. In addition, Healy et al.^44^ recently provided an alternative method that takes phylogenetic relatedness into account, an approach that promises to be useful where reliable proxy data are available.

## Conclusion

Whilst the application of Bayesian isotope mixing models undoubtedly provides robust dietary estimates, this is strengthened by the use of appropriate DTDFs to provide reliable inferences as shown in this study. Experimental studies continue to be central in illuminating not only taxon-specific variations in these factors, but also some underlying biological processes that need consideration. The sensitivity of DTDFs in isotope mixing models, particularly due to potential diet effects, was illustrated in this study, highlighting the need to explore appropriate approaches that provide reliable factors, particularly through empirical experiments. Where experimental determination of DTDFs is not feasible, other studies have suggested several alternative approaches, particularly those that explore the ecological processes that are likely to result in variation in isotopic trophic discrimination among different sources. Evidence from many of these studies points to large uncertainties associated with different diet sources, which posits the likelihood of erroneous inferences from isotope mixing model analyses if these uncertainties are not thoroughly explored. Due to both the sensitivity and central role of DTDFs, it is therefore prudent to consider the use of appropriate factors that act as informative priors in trophic ecology studies.

## Supplementary information

Supplementary Information 1.

Supplementary Information 2.

## Data Availability

Stable isotope experimental data for Oreochromis mossambicus are available on Dryad Digital Repository: 10.5061/dryad.n5tb2rbs3.
