# Supplementary material for: Exploring source differences on diet-tissue discrimination factors in the analysis of stable isotope mixing models
Source: Sci Rep. 2020 Sep 25;10:15816. doi: 10.1038/s41598-020-73019-x (PMC7519091; doi:10.1038/s41598-020-73019-x)
Supplement: Supplementary file 2 — Supplementary Information 2. [file 41598_2020_73019_MOESM2_ESM.docx]

**Supplementary 2**

**Exploring source differences on diet-tissue discrimination factors in the analysis of stable isotope mixing models**

Wilbert T. Kadye^1*^, Suzanne Redelinghuys^1^, Andrew C. Parnell^2^ and Anthony J. Booth^1^

^1^Department of Ichthyology and Fisheries Science, Rhodes University, P.O. Box 94, Grahamstown/Makhanda 6140, South Africa.

^2^Hamilton Institute, Insight Centre for Data Analytics, Maynooth University, Kildare, Ireland.

***Corresponding author:** w.kadye@ru.ac.za

*Parameter estimates using the non-linear least-squares (NLS) approach*

Based on the non-linear least-squares (NLS) approach, the $\delta^{13}C$incorporation into the muscle tissue was best explained by one-compartment models, which had the lowest $AICc$ values (Table A1). The $\delta^{13}C$parameter estimates revealed differences among diets, with $\delta^{13}C_{\infty}$ values being highest for the consumer group 1 (-19.23 $\pm$ 0.08 ‰) followed by consumer groups 2 and 3, and lowest for the consumer group 4 (-21.33 $\pm$ 0.12 ‰) (Table A1). The average residence times were variable, and ranged from approximately 6 to 13 days. In comparison to muscle tissue, $\delta^{13}C$incorporation into fin tissue increased asymptotically for the consumer groups 1 to 3, but decreased over time for the consumer group 4. The $\delta^{13}C$incorporation into fin tissue was best supported by one-compartment models. In general, however, for best supported models, the average residence times for $\delta^{13}C$incorporation into fin tissue was relatively higher, ranging from 25 to 100 days.

The$\delta{}^{15}N$ incorporation into muscle tissue was best supported by one-compartment models in all consumer groups (Table A1). In general, $\delta{}^{15}N$ incorporation into muscle tissue increased asymptotically for consumer groups 1 and 2, whereas consumer groups 3 and 4 showed a relatively slight decrease over time. Thus, the $\delta{}^{15}N$parameter estimates indicated higher $\delta^{15}N_{\infty}$values in consumer groups 1 (10.56 $\pm$ 0.17 ‰) and 2 (10.86 $\pm$ 0.15 ‰) compared to consumer groups 3 (8.93 $\pm$ 0.08 ‰) and 4 (8.51 $\pm$ 0.09 ‰). In addition, the average residence times were higher for consumer groups 1 ($\tau$ $\approx$ 33 days) and 2 ($\tau$ $\approx$ 25 days) than for consumer groups 3 ($\tau$ $\approx$ 13 days) and 4 ($\tau$ $\approx$10 days). For the fin tissue, the $\delta{}^{15}N$ incorporation was characterised by asymptotic increase for consumer groups 1 and 2 groups, and asymptotic decrease for consumer groups 3 and 4. For the best supported models, parameter estimates indicated high $\delta^{15}N_{\infty}$values in consumer groups 1 (11.66 $\pm$ 0.22 ‰) and 2 (11.71 $\pm$ 0.18 ‰), and low values in consumer groups 3 (7.21 $\pm$ 0.20 ‰) and 4 (6.96 $\pm$ 0.23 ‰).

**Table S1:** Parameter estimates based on non-linear least squares for $\delta^{13}C$and $\delta{}^{15}N$ isotope incorporation into muscle and fin tissues of *Oreochromis mossambicus*. The estimated parameters were the average residence time for both one-compartment ($\tau$) and two-compartment ($\tau_{mean}=p\tau_{1}+(1-p)\tau_{2}$), and model comparisons were based on sample size-corrected Akaike’s information criterion ($AICc$) and the difference between the values of the two models ($\Delta_{1-2}$).$-$* indicates that model parameters were indeterminate.

| Isotope | Source | Tissue | One-compartment | $\tau$ | $AICc$ | Two-compartment | $\tau_{mean}$ | $AICc$ |
| --- | --- | --- | --- | --- | --- | --- | --- | --- |
| $\delta^{13}C$ | 1 | Muscle | $-19.2-3.5e^{-\frac{t}{1/0.15}}$ | $6.7$ | $208.6$ | $-19.2-3.5(0.9e^{-\frac{t}{1/0.15}}+0.1e^{-\frac{t}{1/0.02}})$ | $11.7$ | $365.6$ |
|  |  | Fin | $-17.8-3.5e^{-\frac{t}{1/0.03}}$ | $33.3$ | $231.6$ | $-17.4-4.0(0.6e^{-\frac{t}{1/0.03}}+0.4e^{-\frac{t}{1/0.01}})$ | $59.3$ | $388.3$ |
|  | 2 | Muscle | $-20.1-2.6e^{-\frac{t}{1/0.16}}$ | $6.2$ | $204.7$ | $-20.1-2.9(0.6e^{-\frac{t}{1/0.18}}+0.4e^{-\frac{t}{1/0.03}})$ | $16.4$ | $316.5$ |
|  |  | Fin | $-18.0-3.5e^{-\frac{t}{1/0.02}}$ | $50.0$ | $218.6$ | $-17.8-12.5(0.9e^{-\frac{t}{1/0.01}}+0.1e^{-\frac{t}{1/0.01}})$ | $100.0$ | $373.5$ |
|  | 3 | Muscle | $-20.1-2.6e^{-\frac{t}{1/0.16}}$ | $6.3$ | $186.1$ | $-20.1-2.9(0.6e^{-\frac{t}{1/0.18}}+0.4e^{-\frac{t}{1/0.03}})$ | $16.4$ | $343.0$ |
|  |  | Fin | $-18.6-3.1e^{-\frac{t}{1/0.04}}$ | $25.0$ | $209.9$ | $-18.6-3.1(0.9e^{-\frac{t}{1/0.04}}+0.1e^{-\frac{t}{1/0.02}})$ | $26.3$ | $366.9$ |
|  | 4 | Muscle | $-21.3-1.3e^{-\frac{t}{1/0.08}}$ | $12.5$ | $214.8$ | $-21.3-1.5(0.6e^{-\frac{t}{1/0.09}}+0.4e^{-\frac{t}{1/0.02}})$ | $25.9$ | $371.7$ |
|  |  | Fin | $-23.1+1.8e^{-\frac{t}{1/0.01}}$ | $100.0$ | $230.3$ | $-$* | $-$* | $-$* |
| $\delta{}^{15}N$ | 1 | Muscle | $10.6-1.6e^{-\frac{t}{1/0.03}}$ | $33.3$ | $200.3$ | $10.7-2.8(0.5e^{-\frac{t}{1/0.10}}+0.5e^{-\frac{t}{1/0.02}})$ | $29.2$ | $355.1$ |
|  |  | Fin | $11.7-2.5e^{-\frac{t}{1/0.03}}$ | $33.3$ | $213.9$ | $11.6-2.6(0.8e^{-\frac{t}{1/0.03}}+0.2e^{-\frac{t}{1/0.01}})$ | $48.0$ | $370.8$ |
|  | 2 | Muscle | $10.9-1.9e^{-\frac{t}{1/0.04}}$ | $25.0$ | $203.2$ | $10.7-1.8(0.1e^{-\frac{t}{1/0.06}}+0.9e^{-\frac{t}{1/5.18)}})$ | $2.3$ | $359.2$ |
|  |  | Fin | $11.7-2.4e^{-\frac{t}{1/0.04}}$ | $25.0$ | $210.7$ | $11.7-2.8(0.6e^{-\frac{t}{1/0.04}}+0.4e^{-\frac{t}{1/0.01}})$ | $58.0$ | $367.7$ |
|  | 3 | Muscle | $8.9+0.2e^{-\frac{t}{1/0.08}}$ | $12.5$ | $193.1$ | $8.9+3.3(0.6e^{-\frac{t}{1/0.45}}+0.4e^{-\frac{t}{1/0.97}})$ | $1.8$ | $348.6$ |
|  |  | Fin | $7.2+2.2e^{-\frac{t}{1/0.02}}$ | $50.0$ | $192.8$ | $7.2+2.3(0.8e^{-\frac{t}{1/0.02}}+0.2e^{-\frac{t}{1/0.01}})$ | $61.0$ | $349.7$ |
|  | 4 | Muscle | $8.5+0.6e^{-\frac{t}{1/0.01}}$ | $10.0$ | $206.8$ | $8.5+2.6(0.4e^{-\frac{t}{1/0.25}}+0.6e^{-\frac{t}{1/0.13}})$ | $6.2$ | $362.8$ |
|  |  | Fin | $7.0+2.4e^{-\frac{t}{1/0.03}}$ | $33.3$ | $204.4$ | $6.9+2.5(0.9e^{-\frac{t}{1/0.02}}+0.1e^{-\frac{t}{1/5.83}})$ | $46.1$ | $361.1$ |
